# *Saccharomyces cerevisiae* Essential Genes with an Opi^−^ Phenotype

**DOI:** 10.1534/g3.113.010140

**Published:** 2014-02-20

**Authors:** Bryan Salas-Santiago, John M. Lopes

**Affiliations:** Department of Microbiology, and Molecular Cellular Biology Graduate Program, University of Massachusetts, Amherst, Massachusetts 01003

**Keywords:** phospholipid synthesis, transcription, regulation, yeast, inositol

## Abstract

The overproduction and secretion of inositol (*i.e*., Opi^−^) phenotype is associated with defects in regulation of phospholipid biosynthesis in yeast. Here we report a screen of the essential yeast gene set using a conditional-expression library. This screen identified novel functions previously unknown to affect phospholipid synthesis.

Transcription of the phospholipid biosynthetic structural genes in *Saccharomyces cerevisiae* is regulated by inositol and choline (Carman and Henry 1999; Greenberg and Lopes 1996; Henry *et al.* 2012; Henry and Patton-Vogt 1998; Jesch *et al.* 2005; Paltauf *et al.* 1992; Santiago and Mamoun 2003). Gene expression is maximally repressed in the presence of inositol and choline and derepressed when they are limiting. This regulation requires several transcription factors that when mutated display one of two phenotypes: inositol auxotrophy or overproduction and secretion of inositol (Opi^−^) (Carman and Han 2009; Greenberg and Lopes 1996; Henry *et al.* 2012). Some of these mutants were identified during the last three decades through traditional genetic screens. However, we previously reported a genomic screen of the viable yeast deletion set (VYDS) for Opi^−^ mutants that identified 91 mutants (Hancock *et al.* 2006). Here we report a screen of the essential yeast gene set using a conditional-expression library (Mnaimneh *et al.* 2004).

Well studied regulators of phospholipid biosynthetic genes include the Ino2p:Ino4p activators, the Opi1p repressor, the Ume6p-Sin3p-Rpd3p histone deacetylase complex (HDAC), the SAGA histone acetyltransferase complex, the ISW2, INO80, SWI/SNF chromatin remodeling complexes, and Mot1p (Ambroziak and Henry 1994; Dasgupta *et al.* 2005; Elkhaimi *et al.* 2000; Fazzio *et al.* 2001; Ford *et al.* 2008; Jackson and Lopes 1996; Kadosh and Struhl 1997, 1998; Nikoloff and Henry 1994; Rundlett *et al.* 1996, 1998; Shen *et al.* 2000; White *et al.* 1991). Ino2p and Ino4p belong to a family of basic helix-loop-helix regulatory proteins, which form a heterodimer that binds to a UAS*_INO_* sequence to activate transcription of most phospholipid biosynthetic genes (*e.g*., *INO1*, *CHO2*, and *OPI3* in Figure 1) (Jesch *et al.* 2005; Santiago and Mamoun 2003). The Ume6p-Sin3p-Rpd3p HDAC, the ISW2 and INO80 chromatin remodeling complexes, and Mot1p are global regulators that play a negative role in phospholipid biosynthetic gene expression (Dasgupta *et al.* 2005; Elkhaimi *et al.* 2000; Fazzio *et al.* 2001; Grigat *et al.* 2012; Jackson and Lopes 1996; Kadosh and Struhl 1997, 1998; Rundlett *et al.* 1996, 1998; Shen *et al.* 2000). Opi1p was the first, and to date, the only repressor found that specifically regulates the phospholipid biosynthetic pathway.

**Figure 1:**
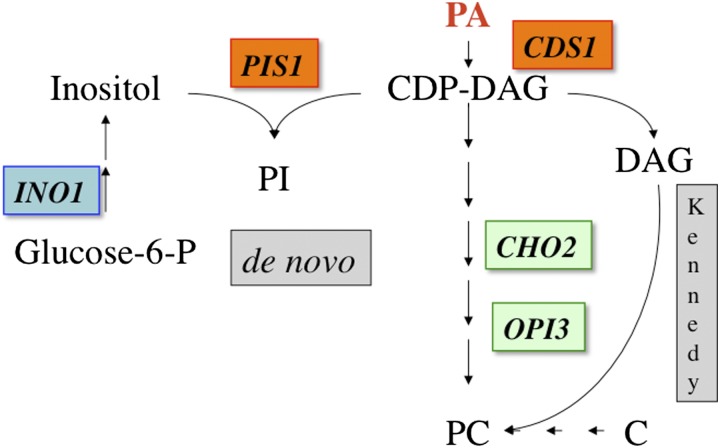
Abridged yeast phospholipid biosynthetic *de novo* and Kennedy pathways. Genes encoding biosynthetic enzymes are italicized and boxed. Those genes noted in green and orange are nonessential and essential (respectively) and yield an Opi^−^ phenotype when mutated. PA, phosphatidic acid; CDP-DAG, CDP-diacylglycerol; PI, phosphatidylinositol; PC, phosphatidylcholine; and C, choline

The *OPI1* locus was first identified in a screen for mutants that overproduce and excrete inositol into the medium in the absence of inositol (Opi^−^ phenotype) (Greenberg *et al.* 1982). The original *opi1* mutant and a small set of similar mutants identified over the next two decades showed that the Opi^−^ phenotype correlated with a defect in repression of the *INO1* gene (Elkhaimi *et al.* 2000; Hirsch and Henry 1986; Hudak *et al.* 1994), which is required for inositol synthesis *de novo* (Figure 1) (Culbertson and Henry 1975). However, most of the 91 Opi^−^ mutants identified in a more recent screen of the VYDS did not affect inositol-mediated repression of an *INO1-lacZ* reporter (Hancock *et al.* 2006).

Our current understanding of the mechanism for inositol-mediated repression of phospholipid biosynthetic gene expression is that it requires translocation of Opi1p from the endoplasmic reticulum (ER) to the nucleus. Repression in response to inositol is actually mediated by the level of phosphatidic acid (PA) (Figure 1). In the absence of inositol, PA levels are elevated and Opi1p binds PA (Loewen *et al.* 2004) and is tethered in the ER by Scs2p, an integral membrane protein (Gavin *et al.* 2002; Kagiwada and Zen 2003; Loewen *et al.* 2003, 2004; Loewen and Levine 2005). When inositol is added, phosphatidylinositol synthesis is increased, causing a decrease in PA levels, and Opi1p is released from the ER. Opi1p rapidly translocates to the nucleus, where it interacts with the Ino2p activator and recruits several HDACs to repress transcription. (Gardenour *et al.* 2004; Grigat *et al.* 2012; Heyken *et al.* 2005; Wagner *et al.* 2001). The addition of choline by itself has little effect on PA levels; however, in combination with inositol, choline further reduces PA levels, resulting in additional repression (Henry and Patton-Vogt 1998). Not surprisingly, blocks in *de novo* phosphatidylcholine (PC) biosynthesis that elevate PA levels also yield an Opi^−^ phenotype (Klig *et al.* 1988; McGraw and Henry 1989; Shen and Dowhan 1996; Summers *et al.* 1988). Thus, *cds1*, *cho2*, and *opi3* mutants all have the Opi^−^ phenotype (Figure 1). The Opi^−^ phenotype of these mutants is conditional and it can be suppressed by adding choline (*i.e.*, C) to the medium. Choline restores PC synthesis through the Kennedy pathway, thereby alleviating the accumulation of PA caused by the block in the *de novo* PC pathway (Figure 1) (Henry and Patton-Vogt 1998).

Consistent with the role of PA as the signal, we reported that reduced expression of the *PIS1* gene (Figure 1) yields an Opi^−^ phenotype (Jani and Lopes 2009). Because *PIS1* is an essential gene, we created a strain harboring a *GAL1-PIS1* gene that allowed us to reduce *PIS1* gene expression by growth in glucose or low galactose concentrations (Jani and Lopes 2009). These growth conditions reduced phosphatidylinositol levels and PA would therefore increase explaining the Opi^−^ phenotype (Jani and Lopes 2009). These results are consistent with another study showing that GFP-Opi1p translocation into the nucleus is slow and impaired in a *pis1* partial function mutant (Loewen *et al.* 2004).

Many studies have shown that screening the VYDS (Giaever *et al.* 2002; Winzeler *et al.* 1999) and an essential yeast mutant gene set (Mnaimneh *et al.* 2004) can yield valuable insight into well-studied processes such as regulation in response to phosphate concentration (Huang and O’Shea 2005). We previously reported the results of a VYDS screen for the Opi^−^ phenotype to further understand repression of phospholipid biosynthesis (Hancock *et al.* 2006). That screen identified all seven of the Opi^−^ mutants that had been identified by several labs over the previous 30 years but also identified 84 new Opi^−^ mutants. Highly represented in this mutant set were the components of the Rpd3p HDAC complex and five of the six nonessential components of NuA4 KAT complex (*EAF1*, *EAF3*, *EAF5*, *EAF7*, and *YAF9*) (Hancock *et al.* 2006). The screen also identified the *reg1* mutant (Hancock *et al.* 2006), which was known to regulate gene expression in response to changes in glucose. Early hypotheses suggested a coordination of glucose use and phospholipid synthesis; however, the mechanism for this coordination was unknown. More recently, it was found that the Opi^−^ phenotype of a *reg1* mutant is actually due to the altered protonation status of PA, as a function of cellular pH, which affects Opi1 translocation to the nucleus (Young *et al.* 2010).

It is well established that phospholipid biosynthesis is coordinated with the unfolded protein response (UPR) and that Opi1p plays a role in this coordination (Betz *et al.* 2002; Cox *et al.* 1997; Jesch *et al.* 2005). The UPR is initiated in the ER in response to accumulation of unfolded proteins (Schröder and Kaufman 2005) and is also induced by depleting inositol (Betz *et al.* 2002; Cox *et al.* 1997). Upon UPR induction, Ire1p is activated initiating splicing of *HAC1* mRNA (Sidrauski and Walter 1997). The spliced *HAC1* transcript produces the Hac1p basic leucine zipper transcription factor that binds to the UPR element of genes such as *KAR2* but also regulates UAS*_INO_* containing promoters by counteracting the function of Opi1p (Cox and Walter 1996). Thus, it was predictable that the VYDS Opi^−^ screen identified genes that are known to affect the UPR (L. C. Hancock and J. M. Lopes, unpublished results). Screening the VYDS for the Opi^−^ phenotype provided a wealth of information about other functions that affect regulation of phospholipid synthesis.

## Materials and Methods

### Strains and growth conditions

This study used the BY4742 (*MAT*α, *his3*Δ1, *leu2*Δ0, *lys2*Δ0, *ura3*Δ1) wild-type and doxycycline (Dox) titratable strains (Giaever *et al.* 2002; Mnaimneh *et al.* 2004; Winzeler *et al.* 1999). The BRS1005 tester strain is a diploid homozygous for the *ino1-13* and *ade1* alleles (Hancock *et al.* 2006). Yeast cultures were grown at 30° in complete synthetic medium (Kelly and Greenberg 1990) containing 2% glucose (w/v) but lacking inositol and choline (I-C-). For the Opi^−^ screen, agarose was reduced to 1.2%, and Dox was added to concentrations noted in the sections to follow.

## Results and Discussion

### Screen of an essential yeast gene library driven by a titratable promoter identifies 122 Opi^−^ mutants

**Figure 2:**
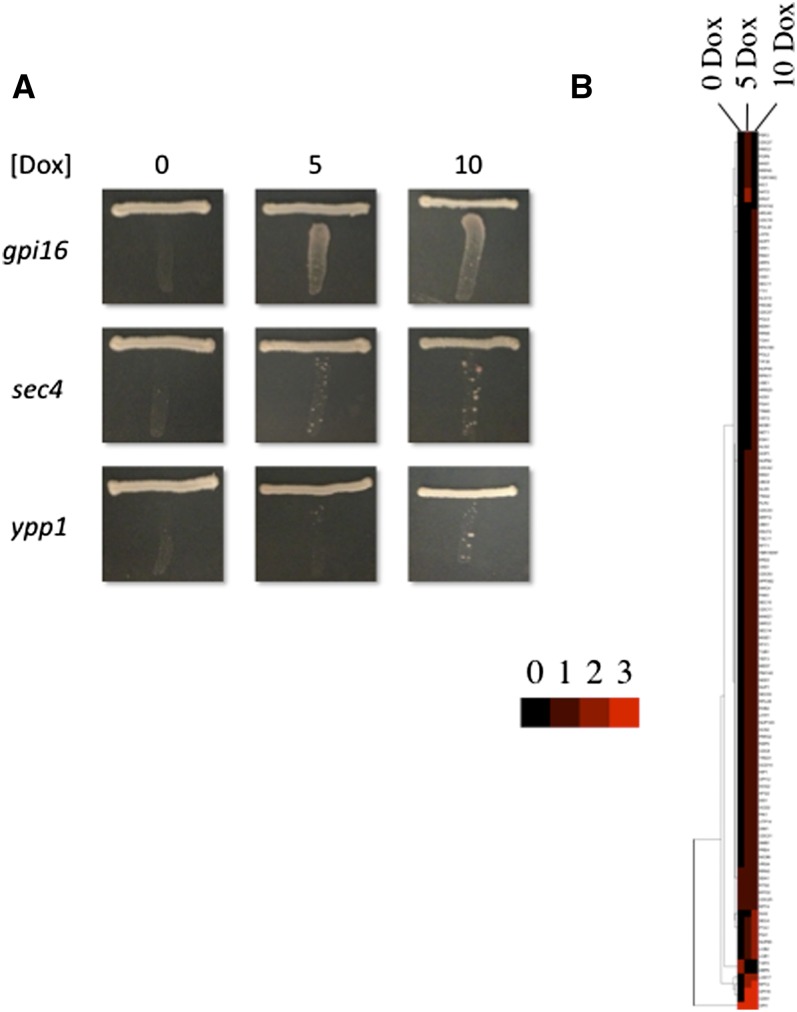
Essential Opi^−^ mutants. (A) Representative Opi^−^ phenotype for the *gpi16* (0,3,3), *sec4* (0,1,2), and *ypp1* (0,0,1) mutants grown under three Dox concentrations. (B) Mutants were clustered with respect to phenotype severity using Cluster 3.0 (http://bonsai.hgc.jp/~mdehoon/software/cluster/software.htm) and displayed using Java Treeview (Saldanha 2004).

To date there had been no screen of the essential genes for defects in phospholipid synthesis, and it is clear that the essential gene set and VYDS are not identical with respect to the biological processes they affect (Winzeler *et al.* 1999). Motivated by this and the success of the VYDS Opi^−^ screen, we conducted a screen of an essential gene library driven by a titratable promoter (Mnaimneh *et al.* 2004). The collection we used contains 838 essential yeast genes driven by a Tet-regulated promoter that is shut off by the addition of Dox. We tested a range of Dox concentrations because different strains have been shown to have differing growth sensitivities (Mnaimneh *et al.* 2004). Our screen of the VYDS for the Opi^−^ phenotype used a pinning strategy (Hancock *et al.* 2006), but this strategy did not work for the essential gene collection. Thus, we used a more laborious but also more sensitive screening assay (Figure 2A) (McGee *et al.* 1994). Briefly, the Tet-driven strain was streaked at the top of plates containing various concentrations of Dox (0, 5, and 10 μg/mL), and lacking inositol and allowed to grow for 1−2 d. A tester strain was then streaked perpendicular to the Tet-driven strain. The tester strain is a diploid homozygous for *ino1* and *ade1* mutants (Swede *et al.* 1992). This strain does not normally grow on media lacking inositol because of the *ino1* mutation. Thus, the Opi^−^ phenotype is observed if the Tet-driven strain secretes inositol into the growth medium allowing the tester to grow. As inositol levels increase in the media, the tester grows more robustly as a red streak (*ade1* phenotype). The tester strain was streaked 3x on each plate and each Tet-driven strain was analyzed in duplicate. The growth of the tester was scored as 0 (no growth), 1, 2, or 3 for progressively varying growth phenotypes. Three researchers independently scored each plate. The screen yielded 122 mutants that all three researchers agreed had a positive test in the two independent assays (Figure 2B and Supporting Information, Table S1). As a control, we included the BY4742 strain (wild type) and an *opi1* mutant, which had an Opi^−^ phenotype under all [Dox]. Sometimes the tester will show a papillar pattern rather than a uniform growth pattern (Figure 2A). These are not revertants or a result of rare mating since the tester is homozygous diploid. We have observed this pattern previously and shown that it correlates with a defect in transcription regulation (Elkhaimi *et al.* 2000; Hancock *et al.* 2006).

Most of the mutant strains did not display an Opi^−^ phenotype in the absence of Dox but did have the phenotype with increasing [Dox] (Figure 2B). In a few cases the Opi^−^ phenotype was observed at lower [Dox] but not at higher [Dox] (top of Figure 2B). This was because the mutant strains did not grow at the higher [Dox]. In a couple of cases the mutant strain yielded an Opi^−^ phenotype in the absence of Dox and did not grow in the presence of Dox (bottom of Figure 2B). These may be false positives or they may result from reduced expression from the Tet promoter (in the absence of Dox) relative to the native promoter and lethality when expression is further reduced by the addition of Dox. As expected, the screen identified the *cds1* mutant which is the only essential gene previously shown to yield an Opi^−^ phenotype (the aforementioned *pis1* allele was not present in the collection) (Klig *et al.* 1988; Shen and Dowhan 1996). The screen also identified five mutants that are duplicated in the collection (*use1*, *cks1*, *rpn11*, *sec4*, and *vrg4*). These results suggest that the screen was successful in identifying legitimate Opi^−^ mutants. We should also note that four of the Opi^−^ mutants (*YNG2*, *HSC82*, *KIC1*, and *SMB1*) are actually not classified as essential in the *Saccharomyces* Genome Database (http://www.yeastgenome.org/). Regardless of this fact, down-regulation did yield an Opi^−^ phenotype so these mutants are retained in our dataset.

### The essential gene and VYDS screens identified mutants in different sets of biological processes

**Figure 3:**
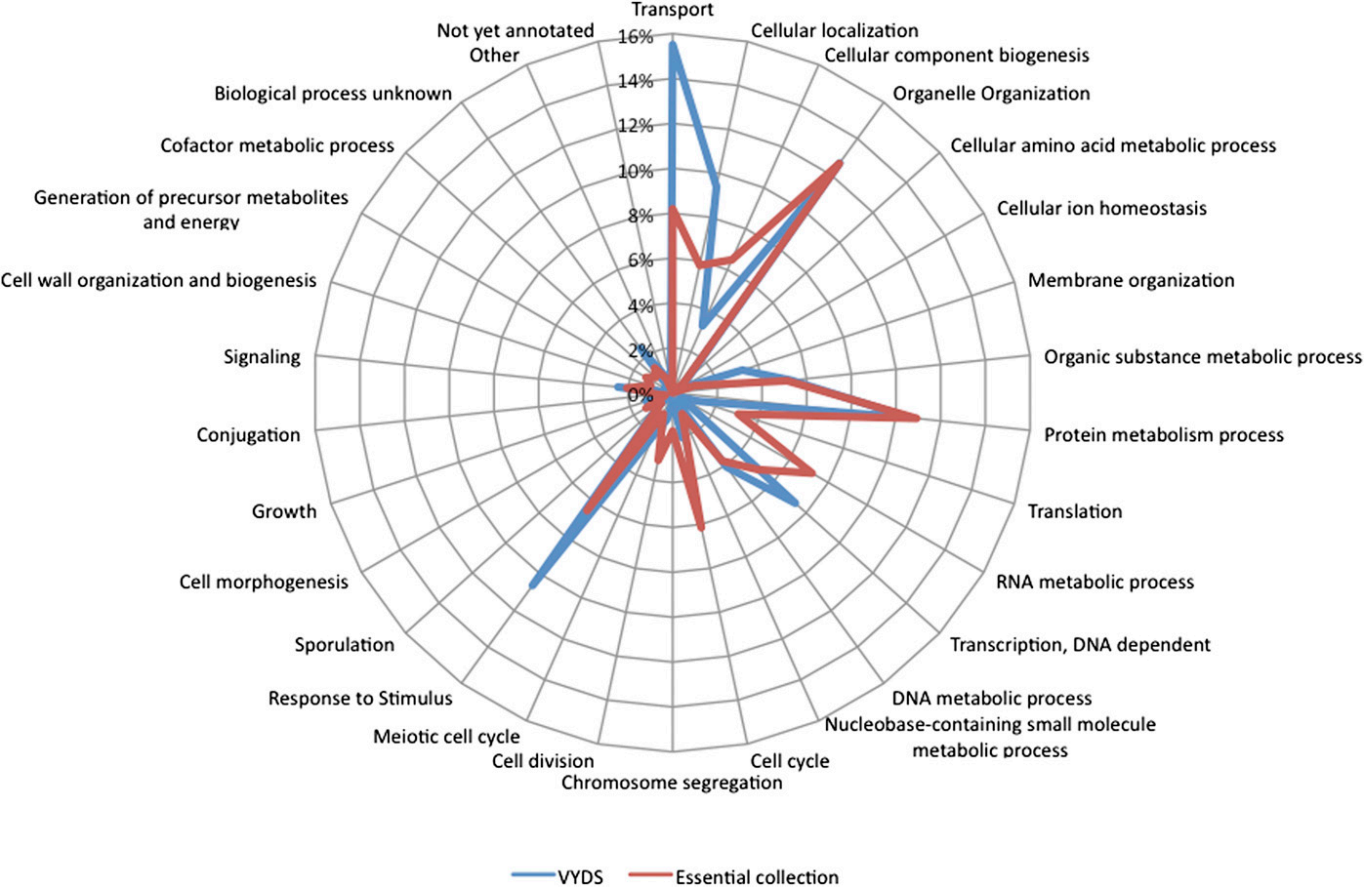
Radar chart comparing percentage of Opi^−^ mutants in different biological processes for the VYDS (blue) and essential (red) mutant collections. Each point on the graph represents the percentage of mutants within each of the Opi^−^ mutant sets in each functional category.

We predicted that the screen might reveal novel processes compared to the VYDS screen. To test this the mutants were grouped based on biological processes using the SGD Yeast Go Slim Mapper software (http://www.yeastgenome.org/cgi-bin/GO/goSlimMapper.pl). The results clearly showed that the two screens yielded different information with respect to biological processes (Figure 3). The essential mutant collection yielded significantly more mutants affecting RNA metabolic processes, the cell cycle, and cell division whereas the VYDS screen identified more mutants in transport, cellular localization, transcription, and response to stimulus.

**Figure 4:**
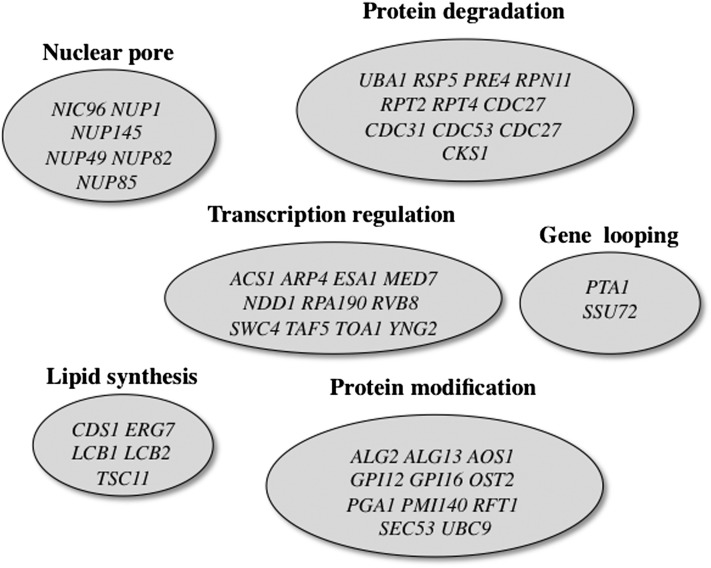
Opi^−^ mutants cluster by functional categories. Shown are those cases in which a significant set of mutants affected a biological function.

Consistent with the results from the VYDS screen and the coordination of phospholipid biosynthesis with the UPR, the current screen identified several mutants that affect protein modifications (Figure 4 and Table S1). These include several genes that glycosylate proteins in the ER (*ALG2*, *ALG13*, *OST2*, *PMI40*, *RFT1*, and *SEC53*). The screen also identified several genes required for synthesis of glycosylphosphatidylinositol anchors (*GPI12*, *GPI12*, and *PGA1*) and for sphingolipid synthesis (*LCB1*, *LCB2*, and *TSC11*) (Figure 4 and Table S1). This is the first report linking these two processes to phospholipid synthesis.

Expression of the *INO1* gene is affected by a mechanism that involves both gene looping and association of the *INO1* promoter with the nuclear pore complex (Brickner 2010; Kerr and Corbett 2010). Interestingly, mutants that affect both gene looping and nuclear pore complex were identified in the Opi^−^ mutant screen (Figure 4 and Table S1). Both the *pta1* and *ssu72* mutants were identified in the essential gene screen. These proteins have been previously shown to be required for gene looping (promoter-terminator) of the *INO1* gene (Ansari and Hampsey 2005). It is not immediately obvious why they should also have an Opi^−^ phenotype but this does provide the first phenotype for gene looping. A significant number of nuclear pore complex mutants (Aitchison and Rout 2012) were identified in the two screens. The VYDS screen identified *NUP84* whereas the essential gene screen identified *NIC96*, *NUP1*, *NUP49*, *NUP82*, *NUP85*, and *NUP145*. On activation, the *INO1* promoter is recruited to the nuclear pore complex via *cis* sequences called DNA Zip Codes (GRS1 and II) within the *INO1* promoter and the adjoining *SNA3* ORF (Ahmed *et al.* 2010; Light *et al.* 2010). Upon transfer to repressing conditions, the *INO1* promoter remains associated with the nuclear periphery for up to three to four generations (Brickner *et al.* 2007). This association is a mechanism for transcriptional memory of recently repressed *INO1* transcription (Brickner *et al.* 2007; Light *et al.* 2010). This memory requires an 11-bp sequence, the memory recruitment sequence, within the *INO1* promoter (Light *et al.* 2010). Importantly, both recruitment to the periphery and transcriptional memory involve distinct mechanisms with different *cis* elements and nuclear pore components, including the Nup1p, Nup84p, Nup145p, and Nic96p subunits (Light *et al.* 2010). Thus, identification of nuclear pore complex mutants in the Opi^−^ screens is consistent with its role in recruiting and regulating the *INO1* promoter.

The essential gene Opi^−^ screen identified several interesting mutants in biological processes that were not identified in the VYDS screen. There was an overrepresentation of mutants in the ubiquitin-mediated degradation pathway (Figure 4 and Table S1). This included the *UBA1* and *RSP5* genes that encode E1 and E3 ubiquitinating enzymes (Kerscher *et al.* 2006). Interestingly, an *rsp5* mutant has been shown to affect expression of an *INO1-lacZ* reporter under derepressing conditions (Kaliszewski *et al.* 2006). The screen also identified several genes required for proteasome function (Forster *et al.* 2010; Tomko and Hochstrasser 2011), including the *PRE4* gene that is required for assembly of the 20S proteolytic core particle; the *RPN11* gene that encodes a deubiquitylase present in the lid of the 19S regulatory particle (Guterman and Glickman 2004); and the *RPT2* and *RPT4* genes that are required for unfolding and translocating the protein substrates as well as opening of the proteasome gate (*RPT2*) (Forster *et al.* 2010; Tomko and Hochstrasser 2011). Another protein modification pathway that was illuminated by the screen is that of an ubiquitin-like modification, SUMO. The screen identified both E1 (*AOS1*) and E2 (*UBC9*) encoding genes (Figure 4 and Table S1) (Johnson 2004; Kerscher *et al.* 2006). This finding is consistent with recent published work showing that a mutation in a deubiquitylation enzyme (*ULP2*) affects *INO1* expression under derepressing conditions by altering the sumoylation status of Scs2p, which normally retains Opi1p in the ER under derepressing conditions (Felberbaum *et al.* 2012).

### Both Opi^−^ screens identified subunits of the NuA4 HAT complex

We previously reported that the VYDS screen identified five of the six nonessential subunits of the NuA4 KAT complex (Hancock *et al.* 2006). The essential collection screen also identified three of the six essential subunits (*ARP4*, *ESA1*, and *SWC4*) (Note: *YNG2* is included in the collection but is not essential.) (Figure 4). One of the essential subunits (*ACT1*) was not present in the collection. Our screen identified *ESA1*, which encodes the KAT activity and contains a chromodomain that interacts with methylated histones as well as *YNG2*, which contains a PHD domain that also interacts with methylated histones (Schulze *et al.* 2010). Thus, both screens collectively identified nine of the possible 12 NuA4 subunits.

It is possible that the proteasome and NuA4 complexes may regulate *INO1* gene expression via a direct role since it has been shown that a 19S proteasome subcomplex works with NuA4 to regulate expression of ribosomal protein genes (Uprety *et al.* 2012). However, the finding that mutations in the 20S complex and the ubiquitin modification pathway yield an Opi^−^ phenotype suggests that protein degradation is the more likely explanation for the phenotype. With respect to the NuA4 complex it is interesting that it functions in activation of gene expression while mutants in other transcription factors that also yield the Opi^−^ phenotype (*e.g.*, *opi1*, *ume6*, *sin3*, and *rpd3*) function in repression (Doyon and Cote 2004; Hancock *et al.* 2006; Schulze *et al.* 2010). In the case of the nonessential Opi^−^ mutants, the mutants yielded elevated expression of the *INO1* target gene under both repressing and derepressing growth conditions, that is, they had a defect in repression (Hancock *et al.* 2006). A trivial explanation for this would be that NuA4 affects repression of *INO1* indirectly by controlling the activation of the *OPI1* repressor gene. However, we found that these mutants did not affect activation of the *OPI1* gene (Hancock *et al.* 2006). Moreover, there is evidence that NuA4 binds the *INO1* promoter (Konarzewska *et al.* 2012). It is also important to note that some of the subunits of the NuA4 complex are shared with the SWR-C complex that is responsible for loading the modified H2A.Z into nucleosomes and H2A.Z is involved in regulation of *INO1* (Lu *et al.* 2009). However, none of the SWR-C−specific components were identified in our screen suggesting that the Opi^−^ phenotype is specific to the NuA4 complex. A more likely explanation is that NuA4 may be acetylating a non-histone regulatory protein that controls *INO1* expression. Consistent with this, an *in vitro* protein acetylation microarray identified many non-histone targets of NuA4 (Lin *et al.* 2009). Along this line it is important that another HAT, Gcn5p, acetylates the Ume6p regulatory protein, which targets it for degradation via the anaphase-promoting complex/cyclosome ubiquitin ligase (Mallory *et al.* 2007, 2012). This occurs as cells are initiating the meiotic program. Consistent with this model the essential gene screen did identify the *CDC27*, which is a component of the anaphase-promoting complex/cyclosome (Figure 4 and Table S1). Although *INO1* is not a meiotic gene, it is regulated by Ume6p and its associated Sin3p/Rpd3 complex (Eiznhamer *et al.* 2001; Elkhaimi *et al.* 2000; Hudak *et al.* 1994; Jackson and Lopes 1996; Kaadige and Lopes 2003; Kadosh and Struhl 1997, 1998). Thus, NuA4 could be regulating *INO1* either through Opi1p or Ume6p via a mechanism that includes protein degradation. Future experiments will address this possibility.

## Supplementary Material

Supporting Information
