# Supplementary material for: Saccharomyces cerevisiae Essential Genes with an Opi− Phenotype
Source: G3 (Bethesda). 2014 Feb 20;4(4):761–7. doi: 10.1534/g3.113.010140 (PMC4059245; doi:10.1534/g3.113.010140)
Supplement: Supporting Information [file supp_4_4_761__index.html]

Saccharomyces cerevisiae Essential Genes with an Opi− Phenotype — Supporting Information 

# *Saccharomyces cerevisiae* Essential Genes with an Opi− Phenotype

## Supporting Information for Salas-Santiago and Lopes, 2014

**Files in this Data Supplement:**

- Table S1 - List of essential genes with an Opi- phenotype. (PDF, 188 KB)
