# Supplementary material for: Saccharomyces cerevisiae Essential Genes with an Opi− Phenotype
Source: G3 (Bethesda). 2014 Feb 20;4(4):761–7. doi: 10.1534/g3.113.010140 (PMC4059245; doi:10.1534/g3.113.010140)
Supplement: Supporting Information [file supp_g3.113.010140_010140SI.pdf]

G3·Genes | Genomes | Genetics

A Screen for *Saccharomyces cerevisiae* Essential Genes with an Opi^{-} Phenotype

Bryan Salas-Santiago and John M. Lopes [1]
Department of Microbiology, and Molecular Cellular Biology Graduate Program, University of Massachusetts, Amherst, Massachusetts 01003

[1]Address for correspondence. University of Massachusetts, 639 North Pleasant St. Amherst, MA 01003. E-mail: jlopes@microbio.umass.edu.

DOI: 10.1534/g3.113.010140

Table S1 List of essential genes with an Opi- phenotype.

Gene	Aliases	Function
AOS1	*RHC31*	Subunit of a heterodimeric nuclear SUMO activating enzyme (E1) with Uba2p; activates Smt3p (SUMO) before its conjugation to proteins (sumoylation)
ACS2		Acetyl-coA synthetase isoform which, along with Acs1p, is the nuclear source of acetyl-coA for histone acetylation; mutants affect global transcription
AFG2	*DRG1*	ATPase of the CDC48/PAS1/SEC18 (AAA) family, forms a hexameric complex; is essential for pre-60S maturation and release of several preribosome maturation factors
ALG13		Catalytic component of UDP-GlcNAc transferase, required for the second step of dolichyl-linked oligosaccharide synthesis; anchored to the ER membrane via interaction with Alg14p
ALG2		Mannosyltransferase that catalyzes two consecutive steps in the N-linked glycosylation pathway
ARC40		Subunit of the ARP2/3 complex, which is required for the motility and integrity of cortical actin patches
ARP4	*ACT3*	Nuclear actin-related protein involved in chromatin remodeling, component of chromatin-remodeling enzyme complexes including NuA4 complex
CDC11	*PSL9*	Component of the septin ring of the mother-bud neck that is required for cytokinesis
CDC19	*PYK1*	Pyruvate kinase, functions as a homotetramer in glycolysis to convert phosphoenolpyruvate to pyruvate
CDC25	*CTN1*	Membrane bound guanine nucleotide exchange factor (GEF or GDP-release factor); indirectly regulates adenylate cyclase through activation of Ras1p and Ras2p by stimulating the exchange of GDP for GTP; required for progression through G1
CDC27	*APC3, SNB1*	Subunit of the Anaphase-Promoting Complex/Cyclosome (APC/C), which is a ubiquitin-protein ligase required for degradation of anaphase inhibitors
CDC31	*DSK1*	Calcium-binding component of the spindle pole body (SPB) half-bridge, required for SPB duplication in mitosis and meiosis II; binds multiubiquitinated proteins and is involved in proteasomal protein degradation
CDC33	*TIF45*	Cytoplasmic mRNA cap binding protein and translation initiation factor eIF4E

CDC37	*SMO1*	Essential Hsp90p co-chaperone; necessary for passage through the START phase of the cell cycle; stabilizes protein kinase nascent chains and participates along with Hsp90p in their folding
CDC42		Small rho-like GTPase, essential for establishment and maintenance of cell polarity; mutants have defects in the organization of actin and septins
CDC53		Cullin, structural protein of SCF complexes (which also contain Skp1p, Cdc34p, Hrt1p and an F-box protein) involved in ubiquitination; SCF promotes the G1-S transition by targeting G1 cyclins and the Cln-CDK inhibitor Sic1p for degradation
CDC8	*MDP1, MUT2, NPI1, UBY1, SMM1*	Thymidylate and uridylate kinase, functions in de novo biosynthesis of pyrimidine deoxyribonucleotides; converts dTMP to dTDP and dUMP to dUTP; essential for mitotic and meiotic DNA replication
CDS1	*CDG1*	Phosphatidate cytidylyltransferase (CDP-diglyceride synthetase); an enzyme that catalyzes that conversion of CTP + phosphate into diphosphate + CDP-diacylglyerol, a critical step in the synthesis of all major yeast phospholipids
CKS1		Cyclin-dependent protein kinase regulatory subunit and adaptor; modulates proteolysis of M-phase targets through interactions with the proteasome; role in transcriptional regulation, recruiting proteasomal subunits to target gene promoters
DBP6		Essential protein involved in ribosome biogenesis; putative ATP-dependent RNA helicase of the DEAD-box protein family
DIM1	*CDH1*	Essential 18S rRNA dimethylase (dimethyladenosine transferase), responsible for conserved m6(2)Am6(2)A dimethylation in 3'-terminal loop of 18S rRNA, part of 90S and 40S pre-particles in nucleolus, involved in pre-ribosomal RNA processing
DOP1		Golgi-localized, leucine-zipper domain containing protein; involved in endosome to Golgi transport, organization of the ER, establishing cell polarity, and morphogenesis
ERD2		HDEL receptor, an integral membrane protein that binds to the HDEL motif in proteins destined for retention in the endoplasmic reticulum; has a role in maintenance of normal levels of ER-resident proteins
ERG7		Lanosterol synthase, an essential enzyme that catalyzes the cyclization of squalene 2,3-epoxide, a step in ergosterol biosynthesis

ESA1	*TAS1*	Catalytic subunit of the histone acetyltransferase complex (NuA4) that acetylates four conserved internal lysines of histone H4 N-terminal tail
GCD14	*TRM61*	Subunit of tRNA (1-methyladenosine) methyltransferase, with Gcd10p, required for the modification of the adenine at position 58 in tRNAs, especially tRNAi-Met
GCD2	*GCD12*	Delta subunit of the translation initiation factor eIF2B, the guanine-nucleotide exchange factor for eIF2; activity subsequently regulated by phosphorylated eIF2
GPI12	*GCR4*	Protein involved in the synthesis of N-acetylglucosaminyl phosphatidylinositol (GlcNAc-PI), the first intermediate in the synthesis of glycosylphosphatidylinositol (GPI) anchors
GPI16		Transmembrane protein subunit of the glycosylphosphatidylinositol transamidase complex that adds GPIs to newly synthesized proteins
HIP1		High-affinity histidine permease, also involved in the transport of manganese ions
HRR25	*KTI14*	Protein kinase involved in regulating diverse events including vesicular trafficking, DNA repair, and chromosome segregation; binds the CTD of RNA pol II
HSC82	*HSP90*	Cytoplasmic chaperone of the Hsp90 family, redundant in function and nearly identical with Hsp82p, and together they are essential
KIC1	*NRK1*	Protein kinase of the PAK/Ste20 kinase family, required for cell integrity possibly through regulating 1,6-beta-glucan levels in the wall; physically interacts with Cdc31p (centrin), which is a component of the spindle pole body
LAS17	*BEE1*	Actin assembly factor, activates the Arp2/3 protein complex that nucleates branched actin filaments; localizes with the Arp2/3 complex to actin patches
LCB1	*END8, TSC2*	Component of serine palmitoyltransferase, responsible along with Lcb2p for the first committed step in sphingolipid synthesis, which is the condensation of serine with palmitoyl-CoA to form 3-ketosphinganine
LCB2	*SCS1, TSC1*	Component of serine palmitoyltransferase, responsible along with Lcb1p for the first committed step in sphingolipid synthesis, which is the condensation of serine with palmitoyl-CoA to form 3-ketosphinganine
LST8		Protein required for the transport of amino acid permease Gap1p from the Golgi to the cell surface; component of the TOR signaling pathway; associates with both Tor1p and Tor2p

MAK21	*NOC1*	Constituent of 66S pre-ribosomal particles, required for large (60S) ribosomal subunit biogenesis; involved in nuclear export of pre-ribosomes
MAS1	*MIF1*	Smaller subunit of the mitochondrial processing protease (MPP), essential processing enzyme that cleaves the N-terminal targeting sequences from mitochondrially imported proteins
MDN1	*REA1*	Huge dynein-related AAA-type ATPase (midasin), forms extended pre-60S particle with the Rix1 complex (Rix1p-Ipi1p-Ipi3p); acts in removal of ribosomal biogenesis factors at successive steps of pre-60S assembly and export from nucleus
MED7		Subunit of the RNA polymerase II mediator complex; associates with core polymerase subunits to form the RNA polymerase II holoenzyme; essential for transcriptional regulation
MGE1	*YGE1*	Mitochondrial matrix cochaperone, acts as a nucleotide release factor for Ssc1p in protein translocation and folding; also acts as cochaperone for Ssq1p in folding of Fe-S cluster proteins
MOB1		Component of the mitotic exit network; associates with and is required for the activation and Cdc15p-dependent phosphorylation of the Dbf2p kinase; required for cytokinesis and cell separation; component of the CCR4 transcriptional complex
MYO1		Type II myosin heavy chain, required for wild-type cytokinesis and cell separation; localizes to the actomyosin ring; binds to myosin light chains Mlc1p and Mlc2p through its IQ1 and IQ2 motifs respectively
MYO2	*CDC66*	One of two type V myosin motors (along with MYO4) involved in actin-based transport of cargos; required for the polarized delivery of secretory vesicles, the vacuole, late Golgi elements, peroxisomes, and the mitotic spindle
NAT2		Protein with an apparent role in acetylation of N-terminal methionine residues
NDD1		Transcriptional activator essential for nuclear division; localized to the nucleus; essential component of the mechanism that activates the expression of a set of late-S-phase-specific genes
NET1	*ESC5, CFI1, SRM8*	Core subunit of the RENT complex, which is a complex involved in nucleolar silencing and telophase exit; stimulates transcription by RNA polymerase I
NIC96		Component of the nuclear pore complex, required for nuclear pore formation; forms a subcomplex with Nsp1p, Nup57p, and Nup49p
NOP7	*YPH1*	Component of several different pre-ribosomal particles; forms a complex with Ytm1p and Erb1p

		that is required for maturation of the large ribosomal subunit; required for exit from G0 and the initiation of cell proliferation
NUP1		Nuclear pore complex (NPC) subunit, involved in protein import/export and in export of RNAs, possible karyopherin release factor that accelerates release of karyopherin-cargo complexes after transport across NPC; potential Cdc28p substrate
NUP49	*NSP49*	Subunit of the Nsp1p-Nup57p-Nup49p-Nic96p subcomplex of the nuclear pore complex (NPC), required for nuclear export of ribosomes
NUP82	*HRB187*	Nucleoporin, subunit of the nuclear pore complex (NPC); forms a subcomplex with Gle2p, Nup159p, Nsp1p, and Nup116p and is required for proper localization of Nup116p in the NPC
NUP85	*RAT9*	Subunit of the Nup84p subcomplex of the nuclear pore complex (NPC), required for assembly of the subcomplex and also for formation of the nucleocytoplasmic Gsp1p concentration gradient that plays a role in nuclear trafficking
NUP145	*RAT10*	Essential nucleoporin, catalyzes its own cleavage in vivo to generate a C-terminal fragment that assembles into the Nup84p subcomplex of the nuclear pore complex, and an N-terminal fragment of unknown function that is homologous to Nup100p
OST2		Epsilon subunit of the oligosaccharyltransferase complex of the ER lumen, which catalyzes asparagine-linked glycosylation of newly synthesized proteins
PAN1	*MDP3, MIP3, DIM2*	Part of actin cytoskeleton-regulatory complex Pan1p-Sla1p-End3p, associates with actin patches on the cell cortex; promotes protein-protein interactions essential for endocytosis
PFY1	*PRF1*	Profilin, binds actin, phosphatidylinositol 4,5-bisphosphate, and polyproline regions; involved in cytoskeleton organization; required for normal timing of actin polymerization in response to thermal stress
PGA1		Essential component of GPI-mannosyltransferase II, responsible for second mannose addition to GPI precursors as a partner of Gpi18p; required for maturation of Gas1p and Pho8p
PGI1	*CDC30*	Glycolytic enzyme phosphoglucose isomerase, catalyzes the interconversion of glucose-6-phosphate and fructose-6-phosphate; required for cell cycle progression and completion of the gluconeogenic events of sporulation
PIK1	*PIK41, PIK120*	Phosphatidylinositol 4-kinase; catalyzes first step in the biosynthesis of phosphatidylinositol-4,5-biphosphate

PLP2		Essential protein that interacts with the CCT (chaperonin containing TCP-1) complex to stimulate actin folding
PMI40		Mannose-6-phosphate isomerase, catalyzes the interconversion of fructose-6-P and mannose-6-P; required for early steps in protein mannosylation
POL2	*DUN2*	Catalytic subunit of DNA polymerase (II) epsilon, a chromosomal DNA replication polymerase that exhibits processivity and proofreading exonuclease activity; also involved in DNA synthesis during DNA repair
POL30		Proliferating cell nuclear antigen (PCNA), functions as the sliding clamp for DNA polymerase delta; may function as a docking site for other proteins required for mitotic and meiotic chromosomal DNA replication and for DNA repair
POL5		DNA Polymerase phi; has sequence similarity to the human MybBP1A and weak sequence similarity to B-type DNA polymerases, not required for chromosomal DNA replication; required for the synthesis of rRNA
POP6		Subunit of both RNase MRP and nuclear RNase P; RNase MRP cleaves pre-rRNA, while nuclear RNase P cleaves tRNA precursors to generate mature 5' ends and facilitates turnover of nuclear RNAs
PRE4		Beta 7 subunit of the 20S proteasome
PRP21	*SPP91*	Subunit of the SF3a splicing factor complex, required for spliceosome assembly
PRP22		DEAH-box RNA-dependent ATPase/ATP-dependent RNA helicase, associates with lariat intermediates before the second catalytic step of splicing; mediates ATP-dependent mRNA release from the spliceosome and unwinds RNA duplexes
PSF2	*CDC102*	Subunit of the GINS complex (Sld5p, Psf1p, Psf2p, Psf3p), which is localized to DNA replication origins and implicated in assembly of the DNA replication machinery
PTA1	*FUN39*	Subunit of holo-CPF, a multiprotein complex and functional homolog of mammalian CPSF, required for the cleavage and polyadenylation of mRNA and snoRNA 3' ends; involved in pre-tRNA processing; binds to the phosphorylated CTD of RNAPII
RFT1		Essential integral membrane protein that is required for translocation of Man5GlcNac2-PP-Dol from the cytoplasmic side to the lumenal side of the ER membrane
RNA1		GTPase activating protein (GAP) for Gsp1p, involved in nuclear transport

RPA190	*RRN1*	RNA polymerase I largest subunit A190
RPL28	*CYH2*	Ribosomal protein of the large (60S) ribosomal subunit; may have peptidyl transferase activity
RPN11	*MPR1*	alloprotease subunit of the 19S regulatory particle of the 26S proteasome lid; couples the deubiquitination and degradation of proteasome substrates
RPT2	*YHS4, YTA5*	One of six ATPases of the 19S regulatory particle of the 26S proteasome involved in the degradation of ubiquitinated substrates; required for normal peptide hydrolysis by the core 20S particle
RPT4	*CRL13, PCS1, SUG2*	One of six ATPases of the 19S regulatory particle of the 26S proteasome involved in degradation of ubiquitinated substrates
RRN3		Protein required for transcription of rDNA by RNA polymerase I; transcription factor independent of DNA template; involved in recruitment of RNA polymerase I to rDNA
RRN5		Protein involved in transcription of rDNA by RNA polymerase I; transcription factor, member of UAF (upstream activation factor) family along with Rrn9p and Rrn10p
RRP45		Exosome non-catalytic core component; involved in 3'-5' RNA processing and degradation in both the nucleus and the cytoplasm
RRS1		Essential protein that binds ribosomal protein L11 and is required for nuclear export of the 60S pre-ribosomal subunit during ribosome biogenesis
RSP5		E3 ubiquitin ligase of the NEDD4 family; involved in regulating many cellular processes including MVB sorting, heat shock response, transcription, endocytosis, and ribosome stability
RTS2		Basic zinc-finger protein
RVB2	*TIP49B, TIH2, TIP48*	Essential protein involved in transcription regulation; component of chromatin remodeling complexes; required for assembly and function of the INO80 complex
SDA1		Highly conserved nuclear protein required for actin cytoskeleton organization and passage through Start, plays a critical role in G1 events, binds Nap1p, also involved in 60S ribosome biogenesis
SEC10		Essential 100kDa subunit of the exocyst complex (Sec3p, Sec5p, Sec6p, Sec8p, Sec10p, Sec15p, Exo70p, and Exo84p), which has the essential function of mediating polarized targeting of secretory vesicles to active sites of exocytosis

SEC11		18kDa catalytic subunit of the Signal Peptidase Complex (SPC; Spc1p, Spc2p, Spc3p, and Sec11p) which cleaves the signal sequence of proteins targeted to the endoplasmic reticulum
SEC14	*PIT1*	Phosphatidylinositol/phosphatidylcholine transfer protein; involved in regulating PtdIns, PtdCho, and ceramide metabolism, products of which regulate intracellular transport and UPR
SEC4	*SRO6*	Rab family GTPase essential for vesicle-mediated exocytic secretion and autophagy; associates with the exocyst component Sec15p and may regulate polarized delivery of transport vesicles to the exocyst at the plasma membrane
SEC53	*ALG4*	Phosphomannomutase, involved in synthesis of GDP-mannose and dolichol-phosphate-mannose; required for folding and glycosylation of secretory proteins in the ER lumen
SIS1		Type II HSP40 co-chaperone that interacts with the HSP70 protein Ssa1p; not functionally redundant with Ydj1p due to due to substrate specificity
SLD5	*CDC105*	Subunit of the GINS complex (Sld5p, Psf1p, Psf2p, Psf3p), which is localized to DNA replication origins and implicated in assembly of the DNA replication machinery
SMB1		Core Sm protein Sm B; part of heteroheptameric complex (with Smd1p, Smd2p, Smd3p, Sme1p, Smx3p, and Smx2p) that is part of the spliceosomal U1, U2, U4, and U5 snRNPs
SOG2		Key component of the RAM signaling network, required for proper cell morphogenesis and cell separation after mitosis
SPP382	*CCF8, NTR1*	Essential protein that forms a dimer with Ntr2p; also forms a trimer, with Ntr2p and Prp43p, that is involved in spliceosome disassembly
SRP21		Subunit of the signal recognition particle (SRP), which functions in protein targeting to the endoplasmic reticulum membrane
SRP72		Core component of the signal recognition particle (SRP) ribonucleoprotein (RNP) complex that functions in targeting nascent secretory proteins to the endoplasmic reticulum (ER) membrane
SSU72		Transcription/RNA-processing factor that associates with TFIIB and cleavage/polyadenylation factor Pta1p; exhibits phosphatase activity on serine-5 of the RNA polymerase II C-terminal domain; affects start site selection in vivo
SUI2		Alpha subunit of the translation initiation factor eIF2, involved in the identification of the start codon; phosphorylation of Ser51 is required for regulation of translation by inhibiting the exchange of GDP for GTP

SWC4	*EAF2, GOD1*	Component of the Swr1p complex that incorporates Htz1p into chromatin; component of the NuA4 histone acetyltransferase complex
TAF5	*TAF90*	Subunit (90 kDa) of TFIID and SAGA complexes, involved in RNA polymerase II transcription initiation and in chromatin modification
TIF35		eIF3g subunit of the core complex of translation initiation factor 3 (eIF3), which is essential for translation; stimulates resumption of ribosomal scanning during translation reinitiation
TOA1		TFIIA large subunit; involved in transcriptional activation, acts as antirepressor or as coactivator
TRM5		tRNA(m(1)G37)methyltransferase, methylates a tRNA base adjacent to the anticodon that has a role in prevention of frameshifting
TRS31		One of 10 subunits of the transport protein particle (TRAPP) complex of the cis-Golgi which mediates vesicle docking and fusion; involved in endoplasmic reticulum (ER) to Golgi membrane traffic
TSC11	*AVO3*	Subunit of TORC2 (Tor2p-Lst8p-Avo1-Avo2-Tsc11p-Bit61p), a membrane-associated complex that regulates actin cytoskeletal dynamics during polarized growth and cell wall integrity; involved in sphingolipid metabolism
TTI1		Putative protein of unknown function; subunit of the ASTRA complex which is part of the chromatin remodeling machinery
TUB1		Alpha-tubulin; associates with beta-tubulin (Tub2p) to form tubulin dimer, which polymerizes to form microtubules
UBA1		Ubiquitin activating enzyme (E1), involved in ubiquitin-mediated protein degradation
UBC9		SUMO-conjugating enzyme involved in the Smt3p conjugation pathway; nuclear protein required for S- and M-phase cyclin degradation and mitotic control; involved in proteolysis mediated by the anaphase-promoting complex cyclosome (APCC)
USE1	*SLT1*	Essential SNARE protein localized to the ER, involved in retrograde traffic from the Golgi to the ER; forms a complex with the SNAREs Sec22p, Sec20p and Ufe1p
UTP14		Subunit of U3-containing Small Subunit (SSU) processome complex involved in production of 18S rRNA and assembly of small ribosomal subunit
UTP7	*KRE31*	Nucleolar protein, component of the small subunit (SSU) processome containing the U3 snoRNA

		that is involved in processing of pre-18S rRNA
VAS1		Mitochondrial and cytoplasmic valyl-tRNA synthetase
VRG4	*VAN2, GOG5, LDB3, VIG4*	Golgi GDP-mannose transporter; regulates Golgi function and glycosylation in Golgi
YEF3	*TEF3*	Gamma subunit of translational elongation factor eEF1B, stimulates the binding of aminoacyl-tRNA (AA-tRNA) to ribosomes by releasing eEF1A (Tef1p/Tef2p) from the ribosomal complex; contains two ABC cassettes; binds and hydrolyzes ATP
YNG2	*NBN1, EAF4*	Subunit of the NuA4 histone acetyltransferase complex that acetylates histone H4 and H2A
YPP1		Cargo-transport protein involved in endocytosis; interacts with phosphatidylinositol-4-kinase Stt4
YBR190w		Dubious open reading frame
YGR190c		Dubious open reading frame
